# Lac Vinum Infantum: A Review of the Work of Infant Milk Depots

**Published:** 1904-09

**Authors:** J. M. Fortescue-Brickdale

**Affiliations:** Registrar and Pathologist to the Bristol Royal Hospital for Sick Children and Women


### LAC VINUM INFANTUM:
### A REVIEW OF THE WORK OF INFANT MILK DEPÔTS.

BY

J. M. FORTESCUE-BRICKDALE, M.A., M.D. Oxon.,

*Registrar and Pathologist to the Bristol Royal Hospital for Sick Children and Women.*

THE fact that we are a self-governing people is so often stated that it is in danger of losing its significance. Reforms and improvements are, however, largely undertaken in response to public demand, and thus it is of the first importance that the public mind should be properly instructed on the various questions of the day. In matters of hygiene and public health the medical profession can exercise an almost unlimited educational influence, and it is with a view of calling the attention of the profession to a subject which is now receiving much consideration in other places that the present article has been written. The subject alluded to is the infant death-rate and the means to be taken for its diminution. The limits of space prevent my quoting figures and authorities for the following premises, but they are sufficiently well recognised to render such a proceeding merely of academic interest:—

1. The mortality of children under one year is very high, especially in large towns, and has not decreased proportionately to the general mortality.
2. It is highest during the first three months of life.
3. It is much higher among artificially fed children than among those fed on breast-milk alone.
4. Breast-feeding is becoming less frequent, and for physical and social reasons is not likely to become much commoner at present.
5. The birth-rate shows a marked and steady decrease.

The obvious remedies for this condition of things are, in addition to general measures of sanitary importance, to encourage breast feeding wherever that is possible, and to improve the conditions under which artificially fed children are reared. This will, of course, especially apply to the poorer classes, where ignorance, carelessness, and the "eternal want of pence" make the problem especially grave. Apart from the individual efforts of medical men and others who work among the poor, breast-feeding has been encouraged in France by the "Consultations des Nourrissons" instituted by M. Budin. Excellent in both intention and results, these establishments are necessarily very slow in operation, can affect but a small proportion of the population and are mainly of value from an educational point of view. Infant day nurseries, which in England are entirely charitable institutions, though in France they are mainly State-supported or State-aided, provide a much-needed assistance to mothers who can neither feed nor take care of their infants. They are, however, costly, each child necessitating an expenditure of from five to ten pounds per annum, and they are not applicable to the large number of cases in which the mother can take charge of her child, though unable through ignorance or poverty to feed it correctly.

There remains yet another method of dealing with the care and feeding of infants of the poorer classes, namely the establishment of milk depôts, or as they are fancifully called, "gouttes de lait." These are primarily institutions for the supply of milk, modified to suit the needs of infants, to the poorer classes at a small charge, in cases where the mother is unable to suckle her infant. In France they are mainly carried on under private medical supervision; in Great Britain the few that have been established are worked by the municipal authorities, and are under the control of the Medical Officer of Health. Generally speaking, the arrangements in the British depôts are as follows:—The milk is supplied by a contractor, under very strict conditions as to quality and purity, the authorities having full rights of testing and inspecting the milk at all stages, from the udder of the cow to the door of the depôt. At the depôt it is modified and "sterilised," and sold to applicants in stoppered bottles, each bottle containing enough for one feed, according to the age of the child for whom it is intended. Enough bottles for 24 hours are sold together in a metal case or basket; a rubber teat is provided, which, when the stopper is removed, fits the neck of the bottle, and full verbal and printed instructions are given to the mothers with the milk. In addition to this a staff of lady-inspectors is organised, who visit the mothers, see that the feeding is properly carried out, instruct them as to the care of the children, and in all necessary cases urge them to call in medical advice. Mothers are also specially told that the milk is only intended for children who cannot be fed on the breast. In Liverpool an arrangement is made with the ordinary dairymen by which the milk can be obtained at their shops, while in York the entire distribution is under contract with various shopkeepers.

With regard to the cost of these institutions, I have been able to collate the experience of five different towns. In

Liverpool the initial cost, before the first depôt was opened, amounted to just over £125, and the total expenditure during the four years 1900-1903 has been about £10,500. During the last three years, during which time the depôt has been open, £4,200, roughly, has been realised by the sale of milk, leaving a net deficit of about £6,300. Over 6,000 infants have been supplied during the three years. In Battersea the amount spent on alteration of premises, machinery, &c., since June, 1902, is about £700. The annual deficit is calculated at £150, and at the end of 1902 300 children were being fed, a figure which had increased to 360 by March, 1903. At St. Helens, where the depôt is not very largely supported, the installation originally cost £255. The Corporation pays rent, wages and wear and tear of plant, and the sale of milk brings in £187 (a little more than its original cost) per annum. At Glasgow the estimated original cost was £650. At York the depôt is maintained at the expense of Mr. B. Seebohm Rowntree, and is not a municipal affair. I am much indebted to Mr. Rowntree for a very full account of the working and results of the institution. The initial cost was £250. This included machinery and plant capable of supplying 400 babies, and bottles, &c., for 50, and the annual loss is over £10c per annum. About 300 children have been on the books since the depôt was opened, nearly a year ago, and 70 are now being supplied (August 3rd, 1904).

The milk is sold at the following prices:—

*Liverpool.*—1s. 6d. a week for all ages.

*Battersea.*—1 to 6 months, 1s. 6d. a week; 6 to 12 months, 2s. a week; over 1 year, 2s. 6d. a week. For children living outside the borough 6d. a week extra is charged.

*Glasgow.*—1 to 3 months, 1s. 2d. a week; 3 to 6 months, 1s. 5½d. a week; 6 to 8 months, 1s. 9d. a week; over 9 months, 2s. 4d. a week.

*York.*—1 to 6 months, 1s. 6d. a week; 7 to 8 months, 1s. 9d. a week; over 8 months, 2s. a week.

The Battersea Corporation pay 9½d. a gallon for milk and 1s. 4d. a pint for cream; in York the milk costs 9d. a gallon in summer and 10d. in winter, and cream is the same price as in Battersea. It is impossible from the figures given to calculate the cost per child per annum, as the length of attendance varies so greatly in different cases.

I will now proceed to deal with the results of these undertakings, as far as they can be estimated. Statistical evidence is notoriously misleading, and that adduced both for and against milk depôts forms no exception to this rule. One method is to compare the infant mortality per 1,000 births for a number of years before and after the establishment of the depôts. Thus Peyroux showed that out of nine French towns where milk depôts had been established the results were unfavourable in three, mediocre in two, a little improved in one, and favourable in only three. Hope showed a marked reduction in infant mortality in Liverpool subsequent to the establishment of the depôts. The average for the three years 1896—1900 was 188 per 1,000 births, in 1901 it was the same, in 1902 and 1903 it fell to 163 and 151 respectively. McCleary in Battersea gives the average for 1897—1901 as 161.8, in 1902 the rate was 136, and in 1903 135. The figures of the Straus Depôt in New York point in the same direction. McCleary, however, has summarised the fallacies involved in this method of comparison. In the first place the number of infants fed at the depôts is too small to influence the death-rate to the degree which the figures seem to indicate; and secondly, other influences such as climatic conditions, epidemic disease, improved sanitation have come into play simultaneously with the establishment of the milk depôts and influenced the death-rate in one or other direction. A more reliable method seems to be to compare the mortality among depôt-fed children with the general mortality of the district among infants under twelve months of age per 1,000 births.

*Liverpool.*—Average death-rate for 1901—1903, 167.3 per 1,000; average death-rate among 4,435 depôt-fed children who were kept under observation, 78 per 1,000.

*Battersea.*—Average death-rate for six months ending December 31st, 1902, 143 per 1,000; average death-rate among 394 depôt-fed children under one year for same period, 98.9 per 1,000. If children dying during first week of life in the borough are deducted from the first average, and fourteen fatal cases where the milk was taken for less than one week omitted from the 394 depôt-fed children, the averages are 118.9 and 63.4 per 1,000.

*St. Helens.*—Average death-rate in borough for 1899—1902, 172.2 per 1,000; average death-rate among depôt-fed children during same period, 98.2 per 1,000. During the first three years children dying after less than one week on the milk are deducted, and in 1902 the period was lengthened to fourteen days. Only children under one year are included.

In considering these figures it must be remembered that in many cases the children were seriously ill before starting the depôt milk, while in others it was only taken for a short time. On the other hand, the figures for the total mortality included breast-fed children. Dr. Ashby, while maintaining a generally friendly but critical attitude towards the milk depôts, refuses to place any confidence in these figures; he points out that the social status of the parents and the length of time during which the milk was taken should be considered, and inquires whether children who only survived their birth a few days are counted as depôt-fed, or placed under the "general mortality" class. As to the first factor, it must be pointed out that the "general mortality" class includes probably all children of good socia status, and the depôt-fed only those of the poorer population: the length of time during which the children were depôt-fed is in two instances allowed for, while in the Battersea figures children surviving for one week are excluded from the general mortality. Mr. Rowntree does not think that the statistics for York are capable of useful quotation, but states that the children kept under observation and regularly weighed have made good progress. Apart, however, from the statistical evidence, there is a very general opinion among medical men there and others who have experience in the districts where milk depôts exist that the results are, on the whole, excellent. In Battersea, in response to a circular from Dr. McCleary, forty medical men reported favourably on the milk, three stated that their experience was not sufficiently large, and only one "found the results disappointing." Park and Holt, in their systematic and extensive researches in New York, found that practitioners there were practically unanimous in their high opinion of the work of the milk depôts.

Criticism is a sound wine, and all new schemes, if they possess any real vitality, are stimulated by it. The general objections raised to the establishment of milk depôts in England must now be considered. These may broadly be divided into (1) socio-political and (2) medical.

1. It has been urged that it is outside the functions of a municipality to distribute "sterilised" and modified milk. Now to determine exactly the functions of a municipality would at the present time be a difficult matter. What is certain is that the general conception of the duties of local authorities is undergoing a process of expansion, and that the limits of these duties are apt to be somewhat arbitrarily laid down in special cases to suit the preconceived opinions of this or that individual. It is generally conceded that the municipality ought to take measures to secure a pure milk supply in the same way that it should secure a pure water supply. If the water supply, though originally pure, became so contaminated by the carelessness of the local water company in its transit from the reservoirs to the houses that it became a source of danger to the people, the local authority would undoubtedly be called upon in no uncertain voice to take the distribution of the water into its own hands and see that it was properly done, even at a large cost to the ratepayers. The analogy between the water supply and the milk supply, though not perhaps exact, because the one affects the entire population and the other only a portion of it—and that a portion which is incapable of lodging an articulate protest—is sufficiently near to be suggestive. It may, of course, be said that, given such stringent regulations as to milk contamination that it can be certain of reaching the consumer in a pure state, all that is necessary is to educate mothers as to the importance of cleanliness and exactitude in feeding their infants. This may be true, but from an educational point of view few methods could be found so effective as the object-lesson provided by a properly conducted milk depôt with its staff of lady inspectors and its living testimony to the good effects of care and intelligence in the matter of artificial feeding. There is, of course, one town in Great Britain where a milk depôt is maintained by private philanthropy, but it is doubtful, at any rate, whether this splendid example will be very extensively followed.

Another objection is that the system tends to "pauperise" the people, an objection which is likely to be largely supported by the vendors of impure milk; but even granting that this view has some definite value, the fact must not be forgotten that it is the problem of infant mortality which has to be solved. Anxiety not to pauperise the parents of the present generation should not allow us to decimate in advance the prospective parents of the next.

2. The medical objections are three in number. It has been particularly urged against milk depôts in France that they tend to discourage breast feeding, and the same thing has been said in England. This is an objection unsupported by figures, and incapable also of statistical disproof. It can only be said that, in this country at any rate, every care is taken to impress upon the mothers that artificial feeding is never to be adopted in preference to breast feeding where the latter is possible; and that among the poorer classes at any rate, where suckling is physically possible and the mother is not compelled to go out all day to work, the extra expense and trouble involved in buying milk at a depôt would certainly act as a deterrent. My own experience, and I believe that of many others, is that women among the poorer classes are generally anxious to suckle their infants, and that when no physical or economic disability exists the difficulty often is to get them to wean their babies at a proper age, breast-fed children two years old being by no means uncommon among hospital out-patients.

The next objection to the British institutions, which has recently been strongly urged by Dr. George Carpenter in the *British Journal of Children's Diseases*, is that they are worked by the municipal authorities, and are not under the direct control of a qualified medical man, as are most of the similar institutions in France. This question is no doubt a very important and difficult one. In the first place, it seems that any strict system such as obtains in France, involving periodical weighing and inspection of the children, would not be practicable in this country, where such methods are not so easily tolerated, although every effort should be made to get reliable records of the results of the system. Next, the position of the medical officer in charge of a milk depôt would, it seems to me, be an untenable one. Either his functions would be limited to keeping the register and supervising the weighing of the infants, in which case it would be scarcely possible to pay him an adequate salary in consideration of the small amount of work he would probably have to perform for some years, or else he would have to be responsible for the general medical treatment of all the infants attending the depôts. The milk depôt would thus become an out-patient department. Prescribing is useless to the poor apart from dispensing. The whole scope and character of the institution would be changed, and in all probability difficulties would arise with local practitioners and hospital authorities. It seems to me that it is much better to trust to the system of home supervision, and to rely on the ordinary medical resources of the locality in cases where more than a suitable diet is required for the welfare of the children. Are the French results better than the English? In the Montmartre district among 384 depôt-fed infants the mortality was 122 per 1,000. The highest I have found in England is 98.2 per 1,000.

The last objection on medical grounds is that the use of "sterilised" milk tends to the production of infantile scurvy. No doubt this is perfectly true; and although, as far as I am aware, only one case has at present been reported in England among depôt-fed infants, others, less well marked perhaps, as Dr. Ashby suggests, have probably occurred. According to Holt's statistics, more than half the cases of this disease are due to the proprietary foods, and less than one-third to "sterilised" milk. Considering the frequency with which the proprietary foods are used by the poorer classes, the risk of infantile scurvy would probably not be increased by the introduction of "sterilised" milk in place of them. Moreover, the practice of boiling infants' milk is by no means uncommon among the poor, at any rate at Bristol; and no one can suppose that once boiled its antiscorbutic properties will be restored by placing it in a dirty bottle to which a long and septic tube is attached. Moreover, the system of house inspection would tend towards the early detection of cases, which would then be brought under medical treatment. To supply a boiled milk is, however, by no means the ultimate ideal of the milk depôt. One such institution in Rochester, N.Y., which has organised its own milk supply, is able to dispense with "sterilisation" altogether, and this should be the object at which other depôts should aim.

In conclusion, I should like to sum up a few points which I think of practical importance in the working of a milk depôt.

1. The co-operation of the medical profession and local hospital authorities is absolutely essential to the success of the undertaking. Out-patient departments and lying-in charities can enormously increase the scope of the milk depôt, and assist in getting proper records kept. All medical men can unite in preventing the depôts being used by those who can suckle their infants, and also in disabusing the parents of the idea that depôt milk is a drug only to be used in desperate cases.

2. Efficient house inspection is hardly less important. Misuse of depôt milk is unfortunately not uncommon, and the general educational influence of well-trained and tactful lady inspectors is very great. It goes without saying that their duties must be clearly defined, and that they must not in any case assume the functions of medical advisers.

3. Sales through local dairy shops should be avoided where possible. No control can be exercised over the customers in these cases, and it is significant that in Liverpool the only complaints as to the milk came from parents supplied in this way. On the other hand, there is no reason why the milk should not be on sale in the better quarters of the town at a price which would be profitable both to the depôt and the middleman. This I believe is about to be done in York.

4. It is probably better to dilute and heat the milk, but the terms "humanised" and "sterilised" should be dropped, as they are inaccurate and misleading. "Modified" and "autoclaved" might be substituted. As Dr. Ashby points out, poor people think "humanised" milk must be as good as "human." And Robertson and Mair have shown that unless milk which has been heated to boiling point for half an hour is kept cool, bacteria which are dangerous to young animals rapidly develop in it.

5. Last, but by no means least, the sources of the milk, if not owned by the municipality, should be under the strictest regulations; and the object should be to obtain a milk so free from contamination that the process of heating can be entirely avoided.

### REFERENCES.

McCleary.—"The Infants' Milk Depôt: Its History and Function," *J. Hygiene*, 1904, iv. 329.

*Ibid.*—"Municipal Milk Depôts," *Brit. M. J.*, 1903, ii. 146.

Mussen.—"Supply of Sterilised Humanised Milk for Infants," *J. State M.*, 1903, xi. 599.

Moore.—Report of M. O. H. of Huddersfield on Infantile Mortality, June, 1904.

Hope.—Report on the Health of the City of Liverpool during 1903. (An abstract appeared in the *Lancet*, 1904, ii. 113.)

*Ibid.*—"Infant Mortality and Milk," *Brit. M. J.*, 1904, i. 784.

Ashby.—"A Case of Scurvy in an Infant fed on Municipal 'Humanised Milk," *Brit. M. J.*, 1904, i. 479. *Vide* also Editorial, *Ibid.*, 503.

Peyroux.—*Lancet*, 1903, i. 1410.

*Ibid.*—*Brit. M. J.*, 1903, i. 95.

*Ibid.*—*Ibid.*, 1904, i. 741.

Variot.—*Ibid.*, 1904, i. 1125.

Carpenter.—*Brit. J. Children's Dis.*, 1904, i. 167.

Robertson and Mair.—*Brit. M. J.*, 1904, i. 1122.

Glasgow.—*Ibid.*, 1903, ii. 1168 (Report).

York.—*Ibid.*, 1903, ii. 1657 (Editorial). *Vide* also *Lancet*, 1904, i. 1220; *Brit. M. J.*, 1903, ii. 469; *Lancet*, 1904, ii. 315; *Brit. M. J.*, 1903, i. 973.

